# The Bladder Microbiome Is Associated with Epithelial–Mesenchymal Transition in Muscle Invasive Urothelial Bladder Carcinoma

**DOI:** 10.3390/cancers13153649

**Published:** 2021-07-21

**Authors:** Wei Tse Li, Anjali S. Iyangar, Rohan Reddy, Jaideep Chakladar, Valmik Bhargava, Kyoko Sakamoto, Weg M. Ongkeko, Mahadevan Rajasekaran

**Affiliations:** 1 Department of Surgery, Division of Otolaryngology-Head and Neck Surgery, UC San Diego School of Medicine, San Diego, CA 92093, USA; wtl008@ucsd.edu (W.T.L.); aiyangar@ucsd.edu (A.S.I.); jchaklad@ucsd.edu (J.C.); rongkeko@health.ucsd.edu (W.M.O.); 2 Research Service, VA San Diego Healthcare System, San Diego, CA 92161, USA; 3 Department of Urology, San Diego VA Healthcare System, University of California, San Diego, CA 92161, USA; roreddy@ucsd.edu (R.R.); kyoko.sakamoto@VA.Gov (K.S.); 4 Division of Cardiology, San Diego VA Healthcare System, University of California, San Diego, CA 92161, USA; vbhargava@health.ucsd.edu

**Keywords:** bladder cancer, BLCA, MIBC, microbiome, intratumor microbiome, EMT, ECM, elastin

## Abstract

**Simple Summary:**

The abundance of microbial species residing within tumors is correlated to cancer progression across many different cancers, including bladder cancer. However, links between the intratumor microbiome of muscle invasive bladder cancer (MIBC) and specific mechanisms of cancer progression have not been well studied. In this paper, we aim to uncover the relationship between microbial abundance in the MIBC intratumor microbiome and epithelial–mesenchymal transition (EMT), one key feature of cancer progression. By comparing the gene expression of EMT-associated genes to the abundance of intratumor microbes in MIBC patients, we found significant correlations between the abundance of microbes and either the upregulation or downregulation of EMT-associated genes. Our findings call for an investigation of possible mechanisms through which the microbiome may regulate EMT in MIBC patients. With further investigation, our findings can be used to provide a new, microbial approach in the diagnosis and therapy of MIBC.

**Abstract:**

The intra-tumor microbiome has recently been linked to epithelial–mesenchymal transition (EMT) in a number of cancers. However, the relationship between EMT and microbes in bladder cancer has not been explored. In this study, we profiled the abundance of individual microbe species in the tumor samples of over 400 muscle invasive bladder carcinoma (MIBC) patients. We then correlated microbe abundance to the expression of EMT-associated genes and genes in the extracellular matrix (ECM), which are key players in EMT. We discovered that a variety of microbes, including *E. coli*, butyrate-producing bacterium SM4/1, and a species of *Oscillatoria*, were associated with expression of classical EMT-associated genes, including E-cadherin, vimentin, SNAI2, SNAI3, and TWIST1. We also found significant correlations between microbial abundance and the expression of genes in the ECM, specifically collagens and elastin. Lastly, we found that a large number of microbes exhibiting significant correlations to EMT are also associated with clinical prognosis and outcomes. We further determined that the microbes we profiled were likely not environmental contaminants. In conclusion, we discovered that the intra-tumoral microbiome could potentially play a significant role in the regulation of EMT in MIBC.

## 1. Introduction

Urothelial bladder carcinoma (UBC) is the tenth most common form of cancer diagnosed globally. In 2020, there were a total of 573,278 new UBC cases and 212,536 deaths caused by UBC [1]. More prevalent in men than women, UBC is the sixth most commonly diagnosed cancer and the ninth leading cause of cancer death in men [1]. Although bladder cancers have various histological origins, approximately 90% of bladder carcinomas arise from the urothelium, the epithelial lining of the urinary bladder [2]. Muscle invasive bladder carcinoma (MIBC) is an advanced stage of UBC, making up to 25% of all cases, in which the carcinoma invades the detrusor muscle underlying the bladder wall [3,4]. MIBC tumors are therefore prone to grow and metastasize rapidly, resulting in poor patient prognosis [5]. 

Epithelial–mesenchymal transition (EMT) is a series of influential molecular mechanisms that promote metastasis in several cancers by increasing cell mobility and decreasing cell–cell adhesion capabilities [5,6]. During EMT, epithelial cells transform into mesenchymal cells, allowing them to detach from the basement membrane and invade nearby tissue. Mesenchymal cells can travel to distant body sites and transition back into epithelial cells in a process known as mesenchymal–epithelial transition, thus initiating metastasis. The prominent role of EMT in MIBC progression has been underscored by studies that demonstrate the upregulation of mesenchymal cell markers, such as N-cadherin and P-cadherin, and a downregulation of epithelial cell markers, including E-cadherin in MIBC tumors [6,7]. 

The remodeling of the extracellular matrix (ECM) surrounding cancer cells plays a role in EMT. There are several mechanisms by which ECM proteins contribute to EMT. For example, matrix metalloproteinases (MMPs), which are proteases found in the ECM, can degrade other ECM proteins, which leads to increased motility of cancer cells [8,9]. Other than MMPs, the ECM contains many proteins, including but not limited to collagens, laminins, and elastin [9,10]. Increased expression of collagens (COL1A1 and COL1A2) has been found to increase cancer progression and decrease survival among UBC patients [11]. Furthermore, laminins (i.e., laminin 332) were also found to play a role in promoting metastasis in UBC by increasing cell motility and invasion [12]. Although the role of elastin in MIBC EMT has not been extensively studied, elastin has been shown to play a significant role in colorectal cancer EMT [13].

Many factors may regulate expression of the genes associated with EMT and the ECM in cancer. Studies investigating EMT in cancer have traditionally focused on pathways intrinsic to cancer cells, but recently, there has been significant interest in the relationship between intra-tumor microbes and EMT [14]. A microbiome has been demonstrated to exist within various human tissues in addition to the gut, where most commensal microbes reside [15]. Importantly, intra-tumor microbe presence has been implicated in the modulation of EMT-associated genes in multiple cancers, including oral cancer, lung cancer, colon cancer, breast cancer, esophageal cancer, and stomach cancer [14].

For decades, the urinary tract and urine were believed to be sterile. However, recent studies challenged this idea, and it is now widely accepted that the urinary tract, including the bladder, is not sterile [16]. The urinary microbiome has since been implicated in UBC [16]. Studies found differences in microbe abundance between urine of healthy patients and UBC patients; however, the mechanisms of how different microbe abundances may interact with EMT and ECM-related protein expression remains unclear [16]. In this study, we correlated the abundance of intra-tumor bacteria to the expression of genes involved in MIBC EMT, including genes encoding for ECM proteins. Additionally, we correlated the expression of microbial species to various clinical variables in order to ascertain whether specific microbes’ presence translates into clinically relevant differences.

## 2. Materials and Methods

### 2.1. Data Acquisition and Download

Raw whole-transcriptome RNA-sequencing data for tumor tissue were downloaded from the TCGA legacy archive (https://portal.gdc.cancer.gov/legacy-archive/search/f, accessed on 5 August 2018) for 405 bladder cancer patients. Level 3 normalized mRNA expression read counts for the above samples were downloaded from the GDC portal (https://portal.gdc.cancer.gov/, accessed on 5 August 2018). Clinical information for all patients were downloaded from the Broad GDAC Firehose (https://gdac.broadinstitute.org/, accessed on 5 August 2018). Genomic alteration information for each patient was obtained from the last analysis report (2016) of the Broad Institute TCGA Genome Data Analysis Center (http://gdac.broadinstitute.org/runs/analyses__latest/reports/, accessed on 5 August 2018). All bladder cancer cases are of the muscle invasive subtype.

### 2.2. Extraction of Microbial Reads and Calculation of Microbial Abundance

Using the Pathoscope 2.0 program, RNA-sequencing data were filtered for bacterial reads via direct alignment through a wrapper for Bowtie2. Bacterial sequences deposited at the NCBI nucleotide database (https://www.ncbi.nlm.nih.gov/nucleotide/, accessed on 9 April 2018) were used. Pathoscope quantifies the relative abundance of each species in each sample, expressed as a percentage. The relative abundance produced were corrected with Aitchison’s log transform to eliminate the effects of compositional data.

### 2.3. Correlation of Microbial Reads to EMT-Related Genes and Pathways

**Figure 1:**
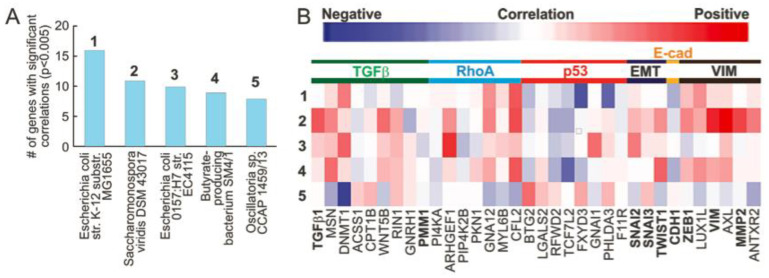
Microbe association with EMT. (**A**,**B**) We correlated microbe abundance to the expression of genes involved in EMT. (**A**). We found that *E. coli*, *S. viridis*, a species of *Oscillatoria*, and a butyrate-producing bacterium are significantly associated with the largest number of EMT/fibrosis-related genes. (**B**). EMT/fibrosis-associated genes were categorized, and correlations are visualized on a heatmap. All of the 5 bacteria species above, except for *Oscillatoria* spp., are generally correlated with increased expression of TGFB-associated genes, RhoA-associated genes, vimentin (VIM)-associated genes, and classic EMT genes (SNAI2, SNAI3, and TWIST), while correlating with decreased expression of p53-related genes and E-cadherin. **C**. GSEA results positively correlate *M. hyopneumoniae* abundance with fibrogenic pathways and negatively correlates *P. mendocina* abundance with these pathways.

Corrected abundance values are correlated with the expression of over 30 EMT-related genes using the Kruskal–Wallis test in R (*p* < 0.05). These genes were found through a literature search and are listed in Figure 1B. They could be classified as TGFB-related genes, RhoA-related genes, p53-related genes, vimentin-related genes, and classical EMT genes. 

### 2.4. Correlations of Microbial Reads to Genes Coding for ECM Proteins and Pathways

Corrected abundance values are correlated with the expression of 275 genes coding for ECM proteins and pathways using the Kruskal–Wallis test in R (*p <* 0.05). These genes were found on the Molecular Signature Database’s (MSigDB) NABA_CORE_MATRISOME gene set [17]. For microbes with non-zero abundance in over 80% of samples (13 microbes), we used gene set enrichment analysis (GSEA) to correlate microbial abundance with ECM-related pathways, including TGFB signaling, collagen pathways, and integrin pathways. Pathways were curated by the Molecular Signature Database (MSigDB, http://www.gsea-msigdb.org/gsea/msigdb/index.jsp, accessed on 26 January 2021).

### 2.5. Correlation of Microbial Reads to Elastin and Elastin-Related Genes

Corrected abundance values are correlated with the expression of elastin (ELN) and elastin-regulating genes using the Kruskal–Wallis test in R (*p <* 0.05). Elastin-related genes are defined as genes upstream of elastin that could influence elastin expression, as determined through a literature search [18,19]. For microbes with non-zero abundance in over 80% of samples (13 microbes), we used GSEA to correlate microbial abundance with two functional gene sets: (1) all genes that are known to upregulate elastin; and (2) all genes that are known to downregulate elastin. 

### 2.6. Assessing the Clinical Relevance of Significant Microbes

Microbes significantly correlated with key genes and pathways above were correlated with clinical variables using the Kruskal–Wallis test in R (*p* < 0.05). We investigated the association between microbe abundance and the presence of a tumor, primary therapy outcome success, a new tumor event after initial treatment (recurrence), residual tumors, neoplasm histological grade, pathologic stages, and vascular invasion of a tumor. In the pathologic T stage analysis, patients with stages T1a and T1b were grouped into stage T1, and likewise for stages T2, T3, and T4. 

### 2.7. Contamination Screening

The abundance of individual microbes in each patient is plotted against total microbe reads in the same patient to determine if any microbe is likely a contaminant. In the resulting scatterplots, if a positive slope exists, it is likely that the microbe was biologically relevant and physically present in the sample, since the counts per microbe increased with the number of microbes sequenced. If the scatterplot has a slope of close to zero (horizontal line), and the counts of all the microbes are substantially above zero, it is likely that the microbe was a contaminant. This reasoning follows from the assumption that similar amounts of microbes will be present regardless of how many microbes are present in the tissue sample if the microbe is an environmental contaminant [17,18]. The Spearman correlation test was used to calculate significance of a linear trendline.

## 3. Results

### 3.1. Microbial Abundance in MIBC Is Significantly Correlated with EMT Related Genes

We downloaded the RNA sequencing data of 405 MIBC patients’ tissue samples from TCGA. RNA expressed by microbial species was computationally quantified using the algorithm Pathoscope 2, which first aligns the RNA sequences to the human genome, discards these reads, and then aligns the remaining sequences to known bacterial genomes. The algorithm outputs the relevant abundances of individual bacterial species within the MIBC tissue samples. By correlating microbial abundance to the expression of genes associated with EMT, we found that the abundance of certain microbes was correlated with the expression of many EMT-associated genes. The microbes that had the greatest number of significant correlations with expression of EMT-related genes were *Escherichia coli str. K-12 substr. MG1655* (16 correlations), *Saccharomonospora viridis DSM 43,017* (11 correlations), *Escherichia coli O157:H7 str. EC4115* (10 correlations), butyrate-producing bacterium SM4/1 (9 correlations), and *Oscillatoria* sp. *CCAP 1459/13* (8 correlations) (Figure 1A). Abundance of all these microbes, with the exception of *Oscillatoria* sp. *CCAP 1459/13*, was positively correlated to genes in the TGFB-, RhoA-, and vimentin-associated genes, as well as the SNAI2, SNAI3, and TWIST1 genes (Figure 1B). Furthermore, these four microbes were negatively correlated to expression of p53-associated genes (Figure 1B). We found that abundance of all but *Escherichia coli O157:H7 str. EC4115* and *Oscillatoria* sp. *CCAP 1459/13* was negatively correlated to E-cadherin expression (Figure 1B). Generally, *Oscillatoria* sp. *CCAP 1459/13* exhibits the opposite correlation trend compared to other microbes (Figure 1B). In summary, a high abundance of several key microbes in MIBC are correlated with EMT markers and EMT-related genes. 

### 3.2. Correlations of Microbial Reads to Genes Coding for ECM Proteins and Pathways

**Figure 2:**
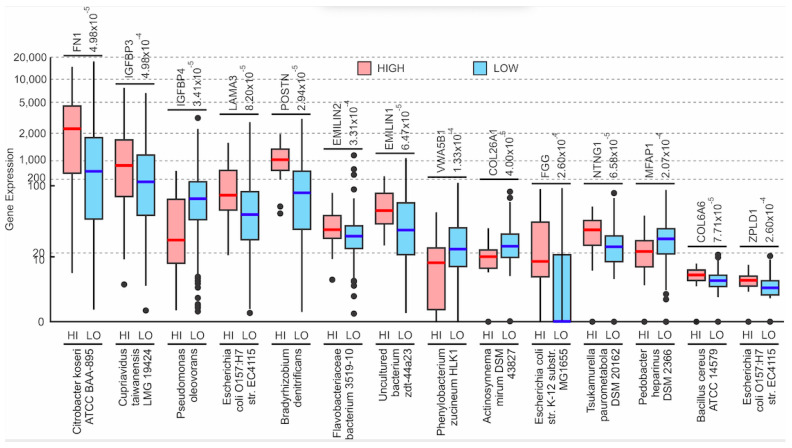
Microbe association with the ECM protein coding genes. Boxplots of specific ECM protein coding gene expression vs. microbial abundance (Kruskal–Wallis test, *p* < 0.05). Dots in the boxplots represent outliers. The cut-off value between high and low microbe abundance for each microbe is listed above the plot for each microbe, beside the name of the gene that the specific microbe is being correlated to. Although these cut-off values were determined from the mean abundance of each microbe across all samples, the values essentially discerned between the presence and the absence of a microbe within a sample.

**Figure 3:**
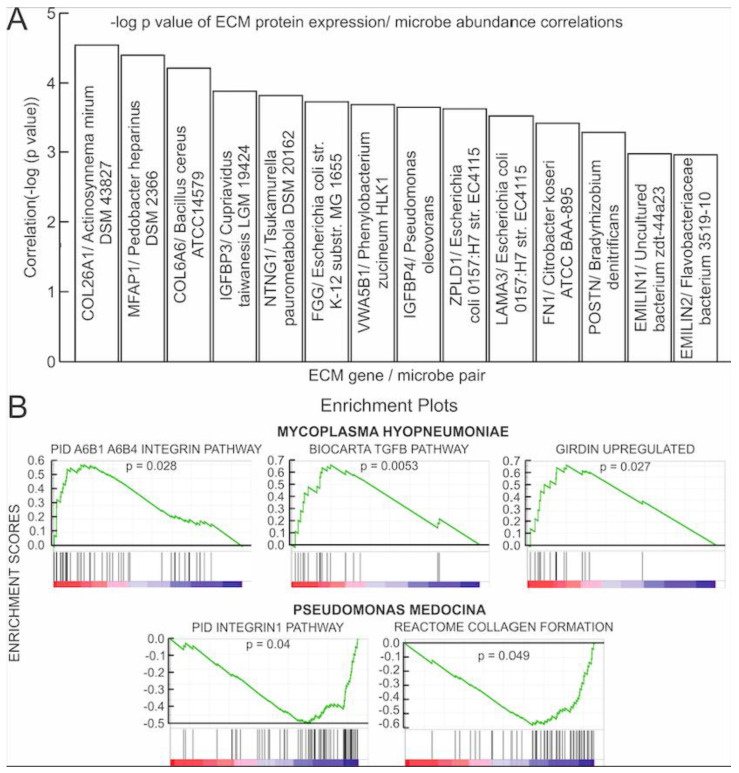
Microbe association with the ECM protein coding genes. (**A**) Bar graph of the Kruskal–Wallis correlation *p*-values. For each significantly correlated ECM gene/microbe pair, the negative logarithm of the *p*-value was plotted. (**B**) Gene set enrichment analysis (GSEA) plots correlating *M. hypopneumoniae* and *P. mendocina* abundance to the expression of a set of genes in ECM pathways. *M. hypopneumoniae* abundance correlated positively to expression of genes in the girdin, integrin, and TGFB pathways. *P. mendocina* abundance correlated negatively with expression of genes in collagen and integrin pathways. A list of genes in each pathway can be found in Table S1A.

The expression of all microbes was correlated with the expression of the 275 hallmark ECM proteins categorized by MSigDB as the NABA_CORE_MATRISOME gene set. The Kruskal–Wallis test found 5307 significant correlations (*p <* 0.05) between microbial abundance and ECM protein gene expression. Of these, two of the most significant correlations were found between abundance of individual microbes and the expression of proteins that are associated with collagens: *Actinosynnema mirum DSM 43827* expression was significantly negatively correlated with COL26A1 expression (*p* = 2.87 × 10^−5^) and *Bacillus cereus ATCC 14579* expression was significantly positively correlated with COL6A6 expression (*p* = 6.15 × 10^−5^) (Figure 2). Other significant correlations were found between individual microbe abundance and expression of the following ECM proteins: microfibrils (MFAP1), IGFBs (IGFBP3 and IGFBP4), netrins (NTNG1), fibrinogens (FGG), Von Willebrand factors (VWA5B1), zona pellucida associated proteins (ZPLD1), laminins (LAMA3), fibronectins (FN1), periostins (POSTN), as well as elastins (EMILIN1 and EMILIN2) (Figure 2). Of these genes, the expression of IGFBP4, VWA5B1, COL26A1, and MFAP1 genes was negatively correlated with the abundance of their corresponding microbes as shown in Figure 2, while the expression of the remaining genes was positively correlated to their corresponding microbe (Figure 2). Notably, *Escherichia coli O157:H7 str. EC4115* abundance was found to be significantly positively correlated with both ZPLD1 (*p* = 0.0002) and the laminin gene LAMA3 (*p* = 0.0003) expression. Figure 3A shows correlations between the other significantly correlated microbe and ECM protein gene pairs, with the -log (*p*-value) as an indicator of significance. 

Using GSEA, we correlated the microbe abundance to known ECM-related pathways or a set of genes with related functions (Table S1A). Only results from microbes found in more than 80% of MIBC patients were presented. We found that abundance of *Mycoplasma hyopneumoniae* is positively correlated with the expression of genes in integrin, girdin, and TGFB pathways, while abundance of *Pseudomonas mendocina* was negatively correlated with expression of genes in ECM-related pathways, including integrin and collagen pathways (Figure 3B). In summary, we found significant association between the ECM pathways and abundance of several microbes, suggesting that microbial presence may influence ECM composition. 

### 3.3. Microbial Expression Is Significantly Correlated to Elastin Expression

**Figure 4:**
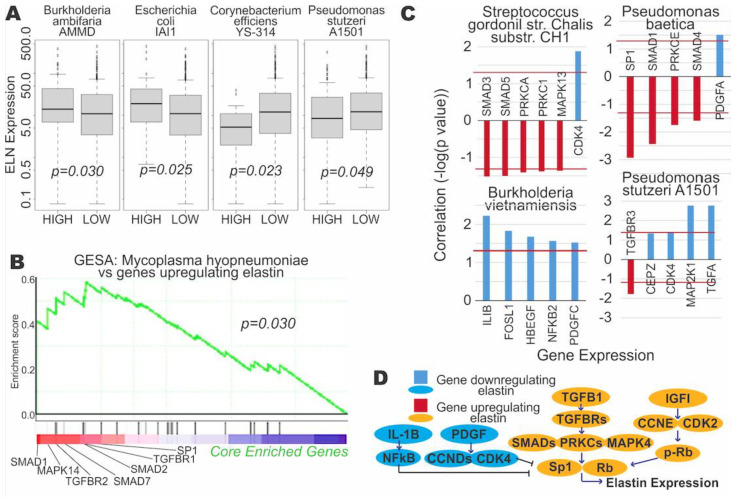
Correlation of microbe abundance to expression of elastin and elastin-regulating genes. (**A**) Boxplots of elastin (ELN) expression vs. microbial abundance (Kruskal–Wallis test, *p* < 0.05). (**B**) Gene set enrichment analysis (GSEA) plot correlating *M. hypopneumoniae* abundance to the expression of a set of genes known to upregulate elastin. The core enriched genes are genes within the entire gene set that contribute the most to a significant positive correlation between the gene set and the microbe. The list of genes upregulating elastin can be found in Table S1A. (**C**) Bar plots of significant correlation between microbial abundance and the genes regulating elastin. Bars pointing upwards from 0 signify a positive correlation between abundance and gene expression, while bars pointing downwards signify a negative correlation. The red horizontal line indicates the significance cutoff (Kruskal–Wallis test, *p* < 0.05). All four microbes shown exhibit an overall association with downregulation of elastin. (**D**) Pathways schematic of the genes regulating elastin expression.

Expression of elastin (ELN) was directly significantly correlated with expression of the four microbes. Elastin is a key protein in the ECM and has recently been implicated in cancer and EMT [13]. We have also discovered a positive correlation between the expression of ELN and the expression of EMT markers, including SNAI1, VIM, and TWIST (Figure S1). ELN expression was correlated positively with *Burkholderia ambifaria AMMD* (*p* = 0.030) and *Escherichia coli IAI1* (*p* = 0.025) expression, while ELN expression was negatively correlated with *Corynebacterium efficiens YS-314* (*p* = 0.023) and *Pseudomonas stutzeri A1501* (*p* = 0.049) expression (Figure 4A). Furthermore, we found significant correlations between microbial expression and genes regulating ELN expression. GSEA determined a significantly positive correlation between abundance of *Mycoplasma hyopneumoniae* and genes upregulating ELN expression (*p* = 0.030) (Figure 3B, Table S1A). Expression of *Streptococcus gordonii str. Challis substr. CH* and *Pseudomonas baetica* were significantly correlated with the expression of many genes involved in upregulation of ELN, while expression of *Burkholderia vietnamiensis* and *Pseudomonas stutzeri A1501* were significantly correlated with expression of genes known to downregulate ELN (Figure 4C). Figure 4D shows the pathways of genes that upregulate and downregulate ELN. It is notable that bacteria from the Pseudomonas genus displayed the most significant correlations between both downregulators and upregulators of ELN expression. Particularly, *Pseudomonas stutzeri A1501* abundance is positively correlated with potent downregulators of ELN expression, such as CEBPZ, CDK4, MAP2K1, and TGFA, which could explain the observed association between higher *P. stutzeri* abundance and lower ELN expression (Figure 4A,C). Additionally, *Pseudomonas baetica* was negatively correlated with the expression of several upstream upregulators of ELN, such as SP1, SMAD1, PKCE, and SMAD4. In summary, we found that several microbe species may regulate the expression of ELN, a key ECM protein potentially involved in EMT. 

### 3.4. Assessing the Clinical Relevance of the Microbes That Are Significantly Correlated to EMT and ECM-Related Genes

**Figure 5:**
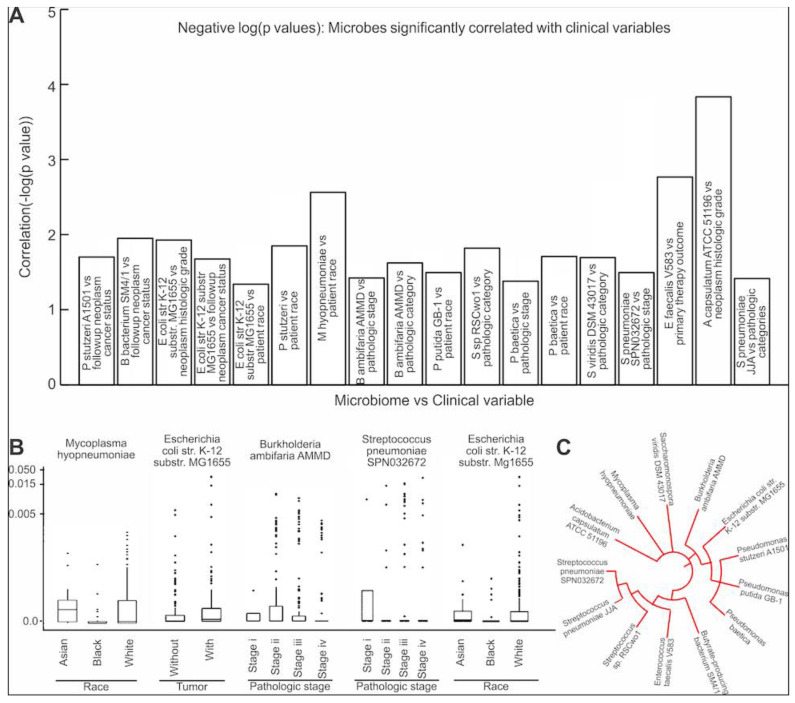
Microbe associations with clinical variables. (**A**) Bar graph of the Kruskal–Wallis correlation *p*-values. For each significantly correlated Clinical variable/microbe pair, the negative logarithm of the *p*-value was plotted. (**B**). Boxplots of specific clinical variables vs. microbial abundance (Kruskal–Wallis test, *p* < 0.05). (**C**) Phylogenic tree for all microbes for which abundance was significantly correlated with clinical variables.

We assessed the clinical relevance of the 25 microbes found to have abundances that were significantly correlated to the expression of the EMT-related genes or ECM protein genes and pathways of elastin (Table S1B). The Kruskal–Wallis test found 18 significant correlations (*p* > 0.05) between microbe abundance and the clinical variables (Figure 5A). The clinical variables that were significantly correlated with abundance of microbes were patient follow-up neoplasm cancer status (correlation with 2 microbes), primary therapy outcome success (correlation with 1 microbe), patient race (correlation with 5 microbes), neoplasm histological grade (correlation with 2 microbes), pathologic stage (correlation with 3 microbes), pathologic N stage (correlation with 3 microbes), and pathologic T stage (1 significant correlation). These clinical variables were correlated with 14 of the 25 unique microbes identified from the previous analyses. We found that the *Pseudomonas* and *Streptococcus* genera were most commonly correlated with the clinical variables (Figure 5C). Notably, *Escherichia coli str. K-12 substr. MG1655* abundance was significantly correlated with three different clinical variables: neoplasm histologic grade (*p* = 0.01), neoplasm cancer status (*p* = 0.02), as well as patient race (*p* = 0.046) (Figure 5B). This suggests a potentially significant role of *Escherichia coli* in the clinical progression of MIBC. The most significant clinical variable correlation was between abundance of *Acidobacterium capsulatum ATCC 51,196* and neoplasm histologic grade (*p* = 0.0001) (Figure 5B). In summary, we found that 14 unique microbes identified to be significantly correlated with EMT or ECM-related proteins are potentially clinically relevant and could influence MIBC prognosis. 

### 3.5. Contamination Screening

Contamination screening was conducted on the 33 microbes that were found to be significant in our analyses (Table S1C). TCGA does not mandate strict control of sterility in tissue storage and sequencing since these samples were not designated for microbiome studies. Therefore, these samples could potentially be contaminated by bacteria from the environment before RNA sequencing. We graphed individual bacterial abundance to total abundance of all bacteria for each sample to evaluate potential contamination (Figure S2). We expect that if a bacterial species was introduced from the environment, it would be similarly abundant across multiple samples regardless of the size of the tissue sampled (reflected in total microbial abundance). This is based on the reasoning that contamination occurs mostly during storage and processing, where samples with different amounts of RNA are stored on the same plates or wells where microbes could reside [19]. Upon comparing the abundance of singular microbes to total microbial abundance with the Spearman correlation test, we found no significant contamination.

## 4. Discussion

In this study, we uncovered significant correlations between intra-tumoral microbial abundance and the expression of EMT-related genes. While previous research has examined the relationship between intra-tumoral microbes and EMT, almost no study examined such a relationship in MIBC or other forms of bladder cancers. The sole study that has explored this topic, to the best of our knowledge, found that urinary *E. coli* isolated from a bladder cancer patient was able to promote EMT in bladder cancer cell lines [20]. However, this study only profiled one bacterial species derived from a single patient. Despite this limitation, its results corroborate our findings, where *E. coli* was among the bacterial species with the greatest number of correlations to different EMT-associated genes. 

Other bacteria that were implicated in EMT in our data include a butyrate-producing bacterium and a member of *Oscillatoria*. Both bacteria release metabolites that are known to influence cancer cells. Butyrate is a powerful short-chain fatty acid that has myriad effects on cancer cells, including induction of apoptosis and reduced proliferation [21]. However, different studies have reported opposite effects of butyrate and butyrate-producing bacteria on cancer. One study found that in ovarian cancer, butyrate induces E-cadherin expression, which could reverse EMT, but also upregulates SNAIL1, an EMT-inducer [22]. In another study, butyrate was found to inhibit E-cadherin in melanoma [23]. In human colon cancer, butyrate-producing bacteria could be either tumor-promoting or anti-tumor, depending on the phenotype of the cancer [24]. In our data, the abundance of butyrate-producing bacteria is positively correlated with the expression of a variety of EMT-promoting genes. On the other hand, the presence of *Oscillatoria* is strongly negatively correlated with the EMT-promoting genes in our data. *Oscillatoria* is a member of the Cyanobacteria, which are not well characterized in humans but have been discovered to be present in the gut [25]. Importantly, members of *Oscillatoria* produce the natural antioxidant butylated hydroxytoluene [26]. This may account for its correlation with reduced EMT, as oxidative stress and the presence of reactive oxygen species have emerged as powerful regulators of ECM proteins and the EMT process [27]. 

In addition to genes classically associated with EMT, gene expression of ECM proteins was also found to correlate with intra-tumoral microbial abundance. ECM proteins are critical to cancer cell invasion and metastasis. In UBC, proteins such as collagens, laminins, fibronectins, etc., are all associated with survival [28]. During cancer cell metastasis and invasion, ECM proteins degrade and integrins rearrange to allow cells to undergo EMT [27,28]. Microbes have been implicated in modulation of the ECM. Many microbes are known to release proteases, especially collagenases, and it is hypothesized that these proteases could influence ECM protein turnover [29]. However, no study has confirmed this hypothesis thus far for bladder cancer. 

Out of the different ECM proteins, we specifically focused on the protein elastin. Elastin is a key protein in the ECM that has not been extensively explored for its potential role in cancer progression. Elastin is commonly known as a structural ECM protein that provides elasticity to tissue. However, it has recently been strongly implicated in colorectal cancer [13]. Increased elastin expression was associated with increased colorectal cancer progression, and the mechanism could be through the induction of EMT [13]. ELN is also involved in the EMT of non-small cell lung cancer [30]. The existing literature does not report any association between ELN and EMT in MIBC. However, we observed a highly significant positive correlation between ELN expression and the expression of EMT markers, including VIM, SNAI1, and TWIST. We believe that ELN could potentially regulate EMT in MIBC as well, and the relationship between ELN and MIBC should be elucidated further in vivo in the future. We observed strong correlation between the abundance of several microbes with elastin expression as well as genes that regulate elastin, suggesting that the intra-tumor microbiome could regulate elastin expression. 

We compared the list of microbes obtained from the TCGA database with microbes that have been discovered in bladder cancer samples through 16s rRNA sequencing. A recent study investigated the bladder cancer microbiome using samples from 43 bladder cancer patients and 10 controls through 16s sequencing [31]. This study found the *Actinomyces*, *Achromobacter*, *Brevibacterium*, and *Brucella* genera to be highly differentially abundant in bladder cancer patients. The TCGA data used in this study also contain Actinomyces, *Achromobacter*, and *Brucella*, but not *Brevibacterium*. Another recent paper published in *Nature* studied the bladder cancer-related microbiome in 24 samples of tissue and urine samples from 10 bladder cancer patients [32]. By sequencing 16s rRNA and performing a comparative analysis of the median fraction total reads at the genus level, this paper listed the 28 genera that were most abundant in both urine and tissue samples. A total of 18 of the 28 genera listed in this study were found in the list of microbes obtained from the TCGA database for our study. Although this shows significant overlap between the data, we acknowledge the differences in the data obtained through TCGA compared to 16 s sequencing data. One reason for the inconsistency in the data is that TCGA exclusively contains samples from patients with MIBC, while the 16s sequenced data from the aforementioned studies contains a mixture of samples from both MIBC and NMIBC patients. This difference may account for some differences in microbe abundances. Other differences may be a result of the resolution level of Pathoscope 2.0, the software used in our study to filter bacterial reads via direct alignment. Specifically, because bacterial genomes may be similar for bacteria in the same taxon, Pathoscope may not be able to distinguish between these bacteria. Therefore, comparing results between 16s rRNA sequencing data and Pathoscope-profiled data at the genus level may not be as accurate as if the comparison was made at a higher taxonomy level, such as phylum. 

The central limitation of our study is the lack of a strict contamination control procedure during processing of the tissue samples, which is an inherent limitation of using TCGA samples for analysis. We addressed this limitation using computational analysis by graphing individual microbe abundance to total microbe abundance for every sample. With existing literature on microbial functions corroborating our correlations, as discussed above, we also have confidence that many of the microbes we profiled are biologically relevant and likely not contaminants. Furthermore, many of the microbial genera we identified, including *Pseudomonas*, *Escherichia*, *Corynebacterium*, and *Streptococcus*, were also commonly identified in urine by studies that presumably carefully controlled contamination [33]. Another limitation of this study is the lack of identification of all bacterial species present in the tissue. Since we are limited to aligning RNA sequencing reads to known bacterial genomes, we could not identify bacteria whose genomes have not been sequenced. However, it is likely that the bacterial species already sequenced are the most common and/or functionally important ones [34].

## 5. Conclusions

In conclusion, this study provides a unique investigation of the potential association between intra-tumor microbes and EMT. We uncovered many correlations that have never been reported before. Since EMT is a critical process in bladder cancer invasion and metastasis, these associations could eventually translate into clinically relevant interventions for MIBC. For example, antibiotics or probiotics could be used to target specific bacterial species in MIBC to lessen the chance of metastasis and improve the prognosis of MIBC. Therefore, further investigations into the functional mechanisms by which intra-tumor microbes could influence EMT are extremely important. 

## Data Availability

All data used in this study is publicly available. Raw whole-transcriptome RNA-sequencing data for tumor tissue were downloaded from the TCGA legacy archive (https://portal.gdc.cancer.gov/legacy-archive/search/f, accessed on 5 August 2018) for 405 bladder cancer patients. Level 3 normalized mRNA expression read counts for the above samples were downloaded from the GDC portal (https://portal.gdc.cancer.gov/, accessed on 5 August 2018). Clinical information for all patients were downloaded from the Broad GDAC Firehose (https://gdac.broadinstitute.org/, accessed on 5 August 2018). Genomic alteration information for each patient was obtained from the last analysis report (2016) of the Broad Institute TCGA Genome Data Analysis Center (http://gdac.broadinstitute.org/runs/analyses__latest/reports/, accessed on 5 August 2018). Bacterial sequences deposited at the NCBI nucleotide database (https://www.ncbi.nlm.nih.gov/nucleotide/, accessed on 9 April 2018) were used.

## Supplementary Materials

The following are available online at https://www.mdpi.com/article/10.3390/cancers13153649/s1, Figure S1: Correlation between ELN Expression and Expression of EMT-related Proteins, Figure S2: Contamination Screening, Table S1: Gene and Microbe Lists.
