# Supplementary material for: The Bladder Microbiome Is Associated with Epithelial–Mesenchymal Transition in Muscle Invasive Urothelial Bladder Carcinoma
Source: Cancers (Basel). 2021 Jul 21;13(15):3649. doi: 10.3390/cancers13153649 (PMC8344975; doi:10.3390/cancers13153649)
Supplement: Supplementary file 1 [file cancers-13-03649-s001.zip › Supplemental/Supplemental Figure Legend.pdf]

Supplemental Figure 1: Contamination screening of significant microbial reads. The total microbial abundance is plotted against individual microbe abundance to screen for reads that contain potential contaminants.

Supplemental Figure 2: Pearson correlation found significant correlation between ELN expression and the expression of the EMT-related proteins VIM, SNAI1, and TWIST1.

Supplemental Table 1: Gene and Microbe Lists. (A) List of genes under each pathway used for GSEA analysis. (B) List of 25 microbes used for comparison with clinical variables. (C) List of 33 variables used for contamination screening.
