# Supplementary figures and images for: The Bladder Microbiome Is Associated with Epithelial–Mesenchymal Transition in Muscle Invasive Urothelial Bladder Carcinoma
Source: Cancers (Basel). 2021 Jul 21;13(15):3649. doi: 10.3390/cancers13153649 (PMC8344975; doi:10.3390/cancers13153649)

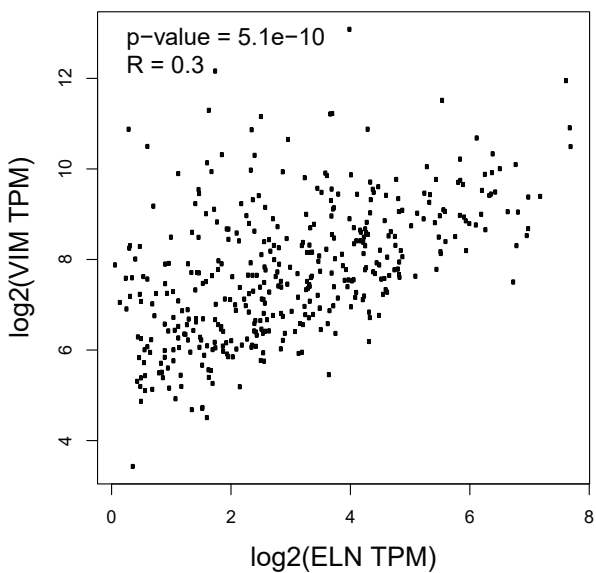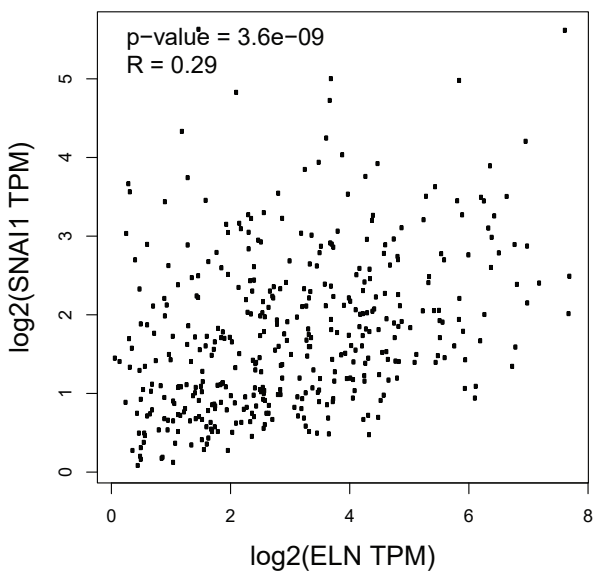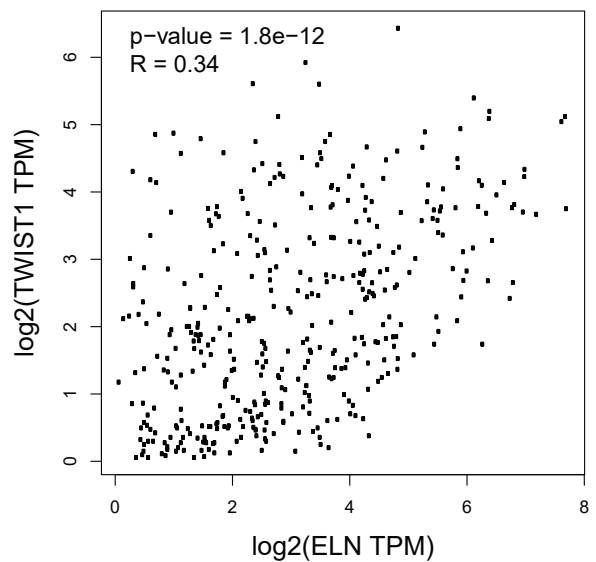

Supplement: Supplementary file 1 [file cancers-13-03649-s001.zip › Supplemental/FigureS1.pdf]

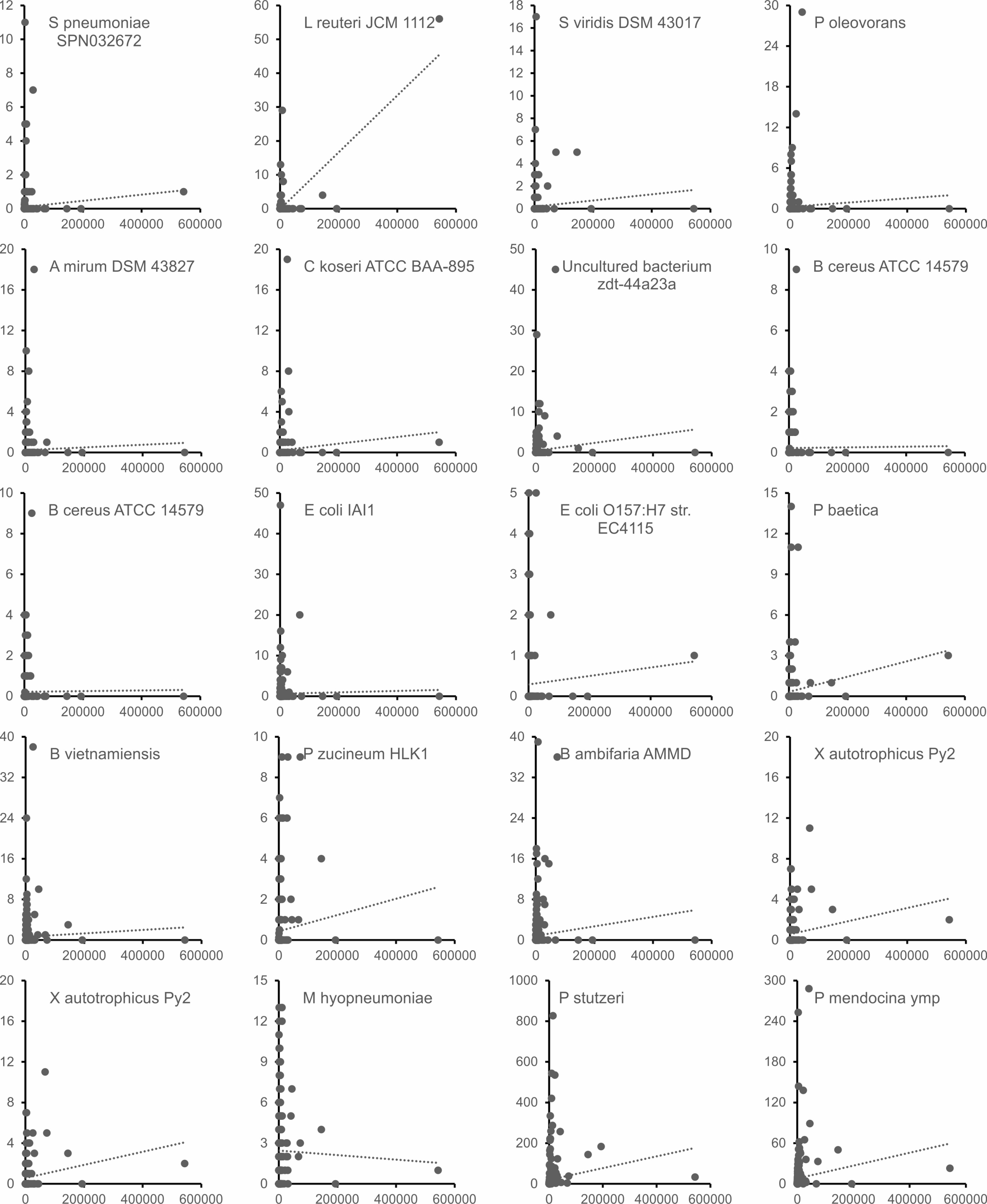

Supplement: Supplementary file 1 [file cancers-13-03649-s001.zip › Supplemental/FigureS2.pdf]
